# The Drug H^+^ Antiporter FgQdr2 Is Essential for Multiple Drug Resistance, Ion Homeostasis, and Pathogenicity in *Fusarium graminearum*

**DOI:** 10.3390/jof8101009

**Published:** 2022-09-26

**Authors:** Tianling Ma, Yiqing Li, Yang Lou, Junrui Shi, Kewei Sun, Zhonghua Ma, Leiyan Yan, Yanni Yin

**Affiliations:** 1 State Key Laboratory of Rice Biology, Institute of Biotechnology, Zhejiang University, 866 Yuhangtang Road, Hangzhou 310058, China; 2 Department of Plant Pathology, Zhejiang Agriculture and Forest University, Hangzhou 311300, China; 3 Ningbo Academy of Agricultural Sciences, Ningbo 315040, China

**Keywords:** H^+^ antiporter (DHA), multiple drug resistance, virulence, Fusarium head blight

## Abstract

Increased emergence of drug resistance and DON pollution pose a severe problem in Fusarium head blight (FHB) control. While the H^+^ antiporter (DHA) family plays crucial roles in drug resistance, the characterization of DHA transporters has not been systematically studied in pathogenetic fungi. In this study, a systematic gene deletion analysis of all putative DHA transporter genes was carried out in *Fusarium graminearum*, and one DHA1 transporter FgQdr2 was found to be involved in multiple drug resistance, ion homeostasis, and virulence. Further exploration showed that FgQdr2 is mainly localized in the cell membrane; its expression under normal growth conditions is comparatively low, but sufficient for the regulation of drug efflux. Additionally, investigation of its physiological substrates demonstrated that FgQdr2 is essential for the transport of K^+^, Na^+^, Cu^2+^, and the regulation of the membrane proton gradient. For its roles in the FHB disease cycle, FgQdr2 is associated with fungal infection via regulating the biosynthesis of virulence factor deoxynivalenol (DON), the scavenging of the phytoalexin, as well as both asexual and sexual reproduction in *F. graminearum*. Overall, the results of this study reveal the crucial roles of FgQdr2 in multiple drug resistance, ion homeostasis, and pathogenicity, which advance the understanding of the DHA transporters in pathogenetic fungi.

## 1. Introduction 

Fusarium head blight (FHB) is one of the most devastating diseases on wheat, barley, maize and other important cereal crops worldwide [1,2]. As one of the main causing agents, filamentous fungus *Fusarium graminearum,* in addition to causing substantial yield losses, is responsible for the serious mycotoxin contaminations of grains. The mycotoxin deoxynivalenol (DON) is the most frequently detected mycotoxin in cereal grains, with a 59% average incidence rate [3]. During the infection of *F. graminearum* in wheats, DON is an important virulence factor, being massively produced and crucial for the spread of this pathogen within hosts [4,5,6,7,8]. Additionally, it can also function as a protein synthesis inhibitor, causing emetic effects, anorexia, immune dysregulation, and teratogenic effects in mammals, which imposes a severe threat to human and animal health [9]. Therefore, DON contamination is closely linked to the FHB disease severity, as well as food security [10,11].

The best way to prevent DON contamination is to manage FHB in the field during crop cultivation. Currently, due to the lack of highly resistant wheat cultivars, the application of chemical fungicides is still the major approach against *F*. *graminearum* [12]. The benzimidazole fungicide carbendazim, as well as the sterol demethylation inhibitor (DMI) fungicides metconazole and prothioconazole, have been widely used for the suppression of FHB over the past three decades [13,14,15]. Owing to their long-term use, carbendazim-resistant and tebuconazole-resistant *F. graminearum* strains have been detected in the fields [16,17,18,19,20]. Moreover, the frequency of fungicide resistance has considerably increased, while the efficacy of the above commonly used fungicides has been significantly reduced in several provinces [13,14,16,17,18,19,20]. Thus, these issues in antifungal drug application pose a severe problem in the treatment of fungal infections. 

In pathogenic fungi, there are mainly four mechanisms associated with the development of antifungal drug resistance: (i) drug target alteration, (ii) drug target overproduction, (iii) drug inactivation, and (iv) reduced uptake and active extrusion [14,21]. The last mechanism is mainly linked to the activation of multidrug efflux pumps—the ATP-binding cassette (ABC) transporter superfamily and the major facilitator superfamily (MFS), which are proposed to actively extrude or compartmentalize drugs and other xenobiotics [22,23,24,25]. The role of the ABC multidrug transporters in antifungal resistance has been well characterized in the past decades [25]. However, much less attention has been paid to the expected role of the MFS transporters in multidrug extrusion [25,26,27,28]. 

As the largest secondary transporter collection, MFS transporters are found in all kingdoms of life, and include millions of sequenced members that are classified into 74 families, with each responsible for a specific substrate type [28,29,30]. MFS transporters involved in drug transport are classified into the drug H^+^ antiporter (DHA) families, including DHA1 and DHA2; the two families differ mainly in the number of transmembrane spans (TMS), with the first having 12, and the second 14 TMS [23,25]. The budding yeast *Saccharomyces cerevisiae* has 12 DHA1 and 10 DHA2 transporters [23]. Interestingly, most of these confer resistance to more than one growth inhibitory compound of particular relevance in the combat against fungal phytopathogens [23,25]. For example, ScFlr1 was found to confer resistance against the agricultural fungicides benomyl [31] and mancozeb [32,33]. ScTpo1-4 and ScQdr3 confer resistance to toxic levels of polyamines, which function as host stress responders [32,34,35]. Additionally, efflux pump ScQdr2 is associated with ketoconazole, quinidine, and barban resistance [25,36,37]. Although DHA transporters are predicted to play important roles in multidrug resistance and cell physiology, they are largely yet to be characterized in pathogenetic fungi. To date, a total of 185 full-size DHA1 proteins and 85 full-size DHA2 proteins have been found in pathogenetic *Candida,* including *Candida albicans*, *Candida glabrata*, *Candida parapsilosis*, *Candida lusitaniae*, *Candida tropicalis*, *Candida guilliermondii*, *Cryptococcus neoformans*, and *Aspergillus fumigatus*; only 5% of the DHA transporters from these organisms have been characterized so far [25]. The predicted involvement of DHA transporters in drug resistance highlights the importance of characterizing them in a systematic way. 

In *F. graminearum*, different from the ABC transporters, which have been systematically identified and characterized in our previous study [38], only one DHA1 transporter FgMfs1 (FGSG_03725) was reported to export salicylic acid, critical for the pathogenicity in *F. graminearum* [39]. As little is known about the other DHA transporters in *F. graminearum*, we therefore were interested in investigating the functions of DHA transporters in *F. graminearum*. In this study, we carried out a systematic gene deletion analysis of all putative DHA transporter genes in *F. graminearum* and found that among 34 DHA transporters, DHA1 transporter FgQdr2 not only regulates multiple drug resistance and ion homeostasis, but also controls virulence via modulating the production of virulence factor DON, phytoalexin export, and asexual and sexual development. These results provide insights into the increasingly severe problems of DON pollution and antifungal drug resistance in FHB control, broadening the knowledge of DHA transporters in fungi. 

## 2. Materials and Methods 

### 2.1. Fungal Strains and Culture Conditions

The wild-type strain PH-1 (NRRL 31084) of *F. graminearum* was used as the parent strain for the transformation experiments. Mycelial growth of the wild-type strain, and the resulting transformants, were assayed at 25 °C on potato dextrose agar (PDA, prepared using 200 g potato, 20 g glucose, and 20 g agar per liter pure water). Conidiation in carboxymethyl cellulose (CMC) liquid medium and induction of sexual development on the carrot agar were performed, as described previously [38]. Each experiment was repeated three times.

### 2.2. Mutant Generation and Complementation, FgQDR2 Overexpression Strain Construction

Constructs for gene deletion in *F. graminearum* using a polyethylene glycol (PEG)-mediated protoplast transformation method were carried out, as described previously [40]. The primers used to amplify the flanking sequences for each gene are listed in Table S1. Putative gene deletion mutants were identified by PCR assays.

To construct the *FgQDR2-GFP* fusion cassette for the complementation, the *FgQDR2* fragment containing the native promoter and ORF (without the stop codon) was amplified with primers listed in Table S1. The resulting PCR products were co-transformed with XhoI digested pYF11-GFP plasmid into *S. cerevisiae* XK1-25 using the alkali-cation yeast transformation kit (MP Biomedicals, Solon, USA). The recombined pYF11-*FgQDR2-GFP* plasmid was extracted from the yeast transformant using a yeast plasmid extraction kit (Solarbio, Beijing, China) and then transferred into *Escherichia coli* strain DH5α for amplification. Then the recombinant plasmid pYF11-*FgQDR2-GFP* was transformed into the ΔFgQdr2 mutant for complementation via the PEG-mediated protoplast transformation method, and the resulting transformant was designated as ΔFgQdr2-C.

The *FgQDR2* overexpression strain were constructed via in situ replacement of the native *FgQDR2* promoter with *gpdA* promoter from *A. nidulans* for constitutive expression. Briefly, a gene replacement cassette carrying the flanking sequences of the *FgQDR2* promoter, hygromycin resistance gene (*HPH*), the coding sequence of the *gpdA* promoter, the GFP-tag coding sequence was constructed using double-joint PCR. Then, the resulting cassette was transformed into the PH-1 strain. The transformants were examined via PCR assays using primers listed in Table S1. The *FgQDR2* ORF upstream of the PCR-confirmed transformants was then sequenced for further examination. Finally, the quantitative reverse transcription PCR (qRT-PCR) and Western blot (WB) analyses were carried out to examine the expression of *FgQDR2* under the *gpdA* promoter.

### 2.3. Quantitative Real-Time Polymerase Chain Reaction (qRT-PCR) Analyses

Total RNA was extracted from the mycelia of each sample using the TaKaRa RNAiso Reagent (TaKaRa Biotechnology Co., Dalian, China). Each RNA sample was reversely transcribed into cDNA with a HiScript II Q RT Kit (Vazyme, R223-01, Nanjing, China). The expression levels of each gene under different culture conditions (as shown in the figure legends) were determined by quantitative real-time PCR with the primers listed in Table S1. For each sample, the quantification of the expression of the *FgACTIN* gene was performed as a reference. The experiment was repeated independently three times.

### 2.4. Western Blotting Assays

*F. graminearum* protein extraction was performed, as described previously [41]. The resulting proteins were separated by 10% sodium dodecyl sulfate-polyacrylamide gel electrophoresis (SDS-PAGE) and transferred to Immobilon-P transfer membrane (Millipore, Billerica, MA, USA). GFP tagged proteins were detected with monoclonal anti-GFP (ab32146, Abcam, Cambridge, UK) antibody; the GAPDH level was detected by the monoclonal anti-GAPDH antibody (Huabio M1306-4) for protein loading reference. Each experiment was repeated three times.

### 2.5. Microscopic Examinations 

The morphology of conidia cultured in CMC liquid medium for 4 d and fresh germinating macroconidia in 2% (*w*/*v*) sucrose solution was observed with a Leica TCS SP5 imaging system (Leica Microsystems, Wetzlar, Germany) after staining with the cell wall-damaging agent calcofluor white (CFW) at 10 μg mL^–1^. Fluorescence signals were examined with a Zeiss LSM780 confocal microscope (Gottingen, Niedersachsen, Germany). The subcellular location of FgQdr2-GFP was exanimated using the following confocal microscopy settings: laser 488 nm at 50% power, pinhole 90 μm, master gain 580. 

### 2.6. Determination of Stress Sensitivity, Virulence, and DON Production

For the determination of sensitivity to various stresses, 5 mm diameter fresh mycelial plugs of each strain were inoculated on PDA, supplemented with or without each stress agent, at 25 °C for 3 d in the dark. The concentration for each supplemented stress agent is indicated in the corresponding figure legends. Each experiment was repeated three times. 

For virulence evaluation on wheat spikelets, a 10 μL aliquot of conidial suspension (10^5^ conidia/mL) was injected into a floret of susceptible wheat (*Triticum aestivum*) cultivar Jimai 22. After 15-d inoculation, infected spikelets in each inoculated wheat head were recorded. For assessing virulence on wheat coleoptiles, the 2 to 3 mm top of the three-day-old coleoptiles was removed, and the wounded top was directly inoculated with a 5 mm diameter fresh mycelial plug. After inoculation, the seedlings were grown in a growth chamber at 25 °C and 95% humidity for later lesion size measurement. To evaluate pathogenicity on corn silks, fresh corn silks were placed on a sterilized filter paper soaked with sterile water. The middle of the corn silks were inoculated with a 5 mm diameter fresh mycelial plug. The infection of the corn silk was measured based on the extent of discoloration after 5 d of incubation at 25 °C. Ten replicates were carried out for each strain. Each virulence experiment was repeated four times.

To determine DON production, the vegetative hyphae of each strain were collected from a 24 h yeast extract peptone dextrose (YEPD, 1% yeast extract, 2% peptone, 2% glucose) culture, then inoculated to TBI liquid medium [42] at 28 °C for 7 d in a shaker (150 rpm) in the dark. Then, the DON production for each sample was extracted and quantified by using a DON Quantification Kit Wis008 (Wise Science, Zhenjiang, China). The dry weight of mycelium was used as an internal reference. 

## 3. Results

### 3.1. Identification of the Drug H^+^ Antiporter (DHA) Families in F. graminearum

The sequences of all the DHA family transporters from *S. cerevisiae*, *C. albicans*, *C. glabrata*, and *A. fumigatus* were used for BLASTP searches to identify their orthologs in the genome of *F. graminearum* (http://www.broadinstitute.org/annotation/genome/fusarium_graminearum/MultiHome.html (accessed on 20 September 2022)). A total of 21 putative DHA1 transporters and 13 putative DHA2 transporters were identified. Conserved domain analysis using the SMART program (http://smart.embl-heidelberg.de) showed that the putative DHA1 transporters contain 9-14 TMSs, and the putative DHA2 transporters contain 10-14 TMSs in *F. graminearum* (Figure S1).

To determine the biological functions of these DHA transporter genes, we performed gene deletion for each of them based on gene homologous recombination using PEG-mediated protoplast transformation. The resulting hygromycin-resistant transformants of each gene were screened by PCR with the primer pairs, as listed in Table S1. All of the 34 DHA transporter genes were successfully knocked out (Figure S2). For each DHA transporter gene, at least three deletion transformants with similar growth phenotypes were obtained. 

### 3.2. The DHA1 Transporter FgQdr2 Is Involved in the Regulation of Multiple Drug Resistance in F. graminearum

**Figure 1:**
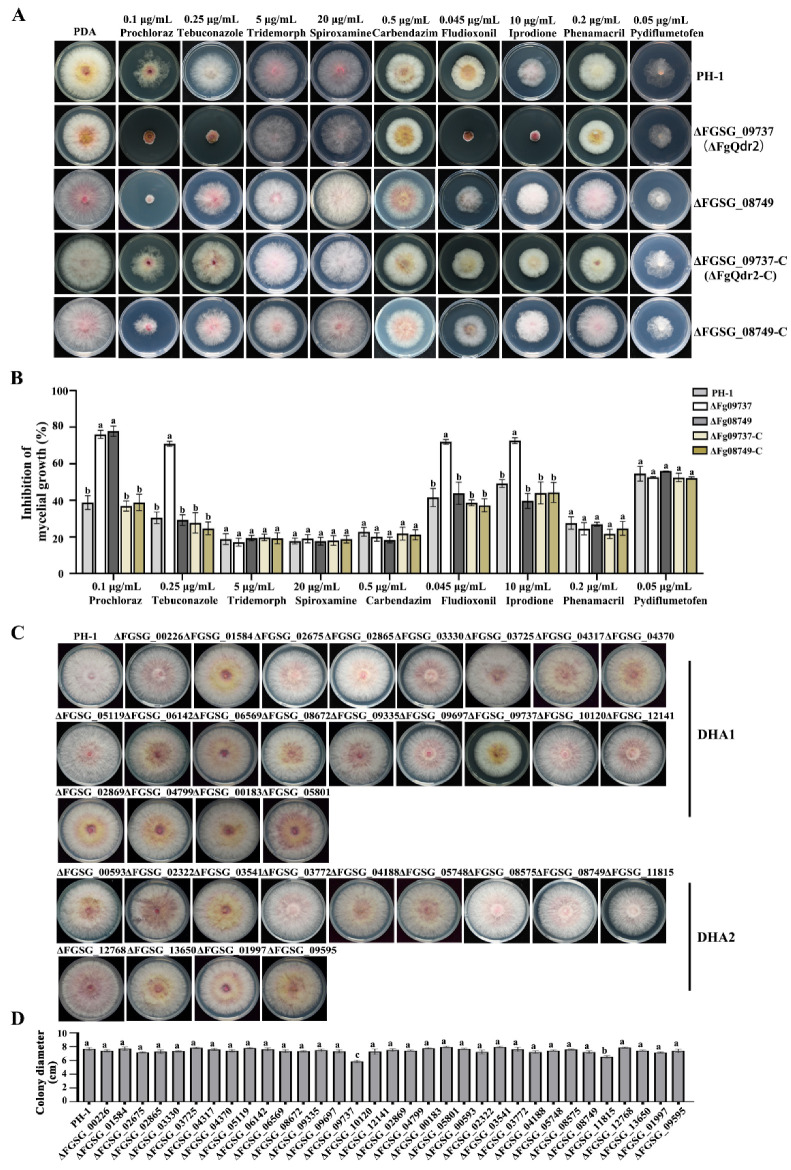
FgQdr2 is required for multiple drug resistance and mycelial growth in *F. graminearum*. (**A**) Sensitivity of each strain to 9 commonly used fungicides. ΔFGSG_09737 (ΔFgQdr2) exhibited increased sensitivity to prochloraz, tebuconazole, fludioxonil, and iprodione, and ΔFGSG_08749 showed increased sensitivity to prochloraz. Each strain was cultured on PDA, and PDA amended with 0.1 ppm prochloraz, 0.25 ppm tebuconazole, 5 ppm tridemorph, 20 ppm Spiroxamine, 0.5 ppm carbendazim, 0.1 ppm fludioxonil, 10 ppm iprodione, 0.2 ppm phenamacril, and 0.02 ppm pydiflumetofen. The images were taken after 3 d incubation at 25 °C, and the mycelial growth inhibition was calculated for each strain. (**B**) The mycelial growth inhibition of each strain under the above drug treatments. (**C**) Colony morphology of PH-1 and 34 DHA transporter deletion mutants. Colony morphology was observed after culture on PDA for 3 d. (**D**) Colony diameters of PH-1 and 34 DHA transporter deletion mutants. For (**B**,**D**), mean and standard deviation were estimated with data from three independent biological replicates (*n* = 3). Different letters indicate significant differences based on ANOVA analysis followed by Turkey’s multiple comparisons test (*p* < 0.05).

To identify the roles of 34 DHA transporters in antifungal drug resistance, the sensitivity of each DHA deletion mutant to nine common fungicides, including prochloraz, tebuconazole, tridemorph, spiroxamine, carbendazim, fludioxonil, iprodione, phenamacril, and pydiflumetofen, was examined. The results showed that the *FGSG_09737* detention mutant (ΔFGSG_09737) exhibited increased sensitivity to prochloraz, tebuconazole, fludioxonil, and iprodione, but not to other fungicides (Figure 1A,B, Table S2). The *FGSG_08749* detention mutant (ΔFGSG_08749) exhibited increased sensitivity to prochloraz, but not to the other fungicides (Figure 1A,B, Table S2), while the rest of the 32 DHA deletion mutants showed no significant difference in antifungal drug resistance compared to the wide type strain PH-1 (Table S2). Additionally, the characterization of mycelial growth and colony morphology in 34 DHA deletion mutants found that only ΔFGSG_09737 and ΔFGSG_11815 exhibited reduced growth, while the other DHA deletion mutants showed neither severe growth deficiency nor colony morphologic differences in comparison with PH-1 (Figure 1C,D). Phylogenetic analysis showed that FGSG_09737 is homologous to Qdr2 in budding yeast and other fungi (Figure S3); a member of DHA1 proteins, FGSG_09737 was therefore designated as FgQdr2. To confirm that phenotypic changes observed in the mutant were due to the deletion of *FgQDR2*, the *FgQDR2* deletion mutant was complemented with a full-length wild-type *FgQDR2* fused with a GFP tag (Figure 1A,B and Figure S4A), and the resulting complemented strain was designated as ΔFgQdr2-C (ΔFgQdr2::Qdr2-GFP). The complemented strain ΔFgQdr2-C was recovered for antifungal drug sensitivity as well as growth (Figure 1A,B). Taken together, these results clearly indicated that the DHA1 transporter FgQdr2 plays important roles in the regulation of multiple drug resistance in *F. graminearum*.

### 3.3. Overexpressed FgQdr2 Is Localized in the Cell Membrane, but Not Involved in Elevated Drug Resistance in F. graminearum

**Figure 2:**
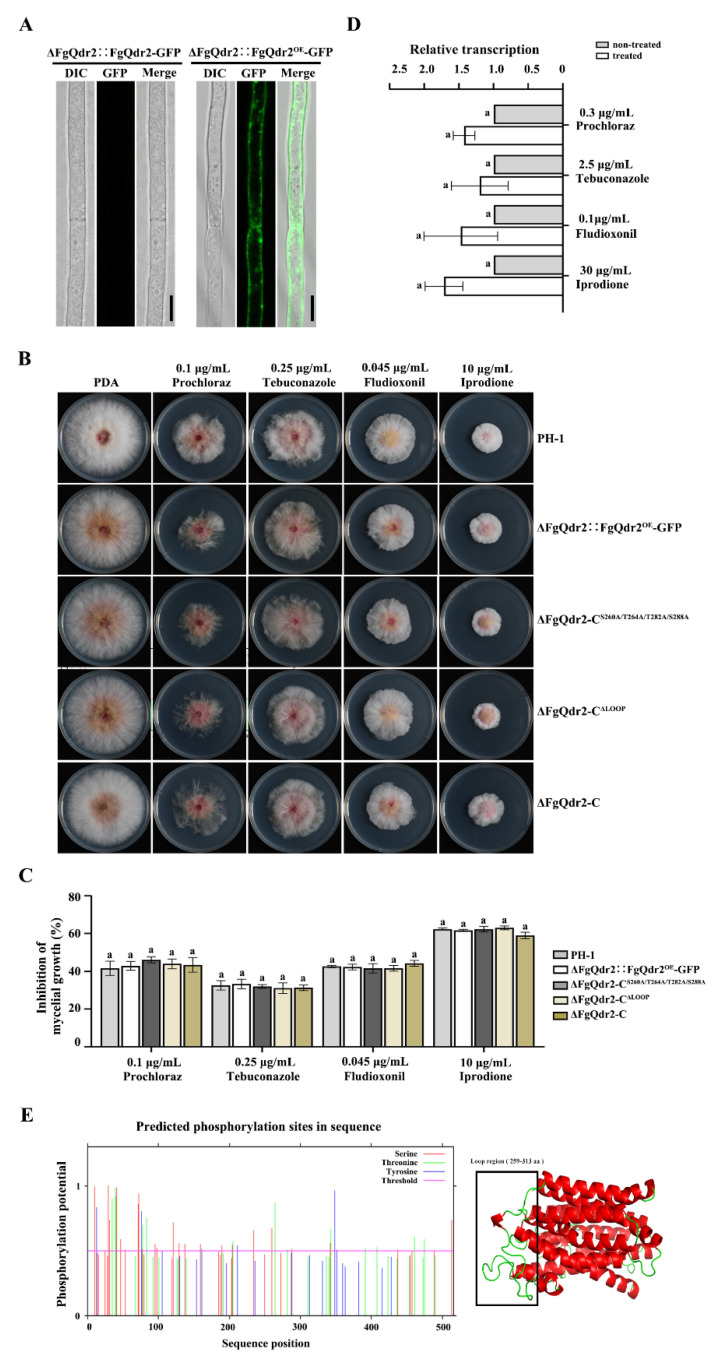
The overexpression, mutation of phosphorylation sites, and deletion of the LOOP region did not alter the regulation of FgQdr2 in drug resistance. (**A**) Overexpressed FgQdr2 is localized along the plasma membrane. Bar = 10 μm. (**B**,**C**) Sensitivity of each strain to fungicide stress, colony morphology (**B**) and mycelial growth inhibition (**C**) were photographed and calculated after 3 d incubation at 25 °C, respectively. (**D**) The quantitative reverse transcription PCR (qRT-PCR) revealed that the transcription level of *FgQDR2* was not upregulated under the above drug treatments. The stain was first cultured in yeast extract peptone dextrose (YEPD) for 24 h, then transferred to YEPD supplied with (treated) or without (non-treated) the corresponding fungicide. The *FgQDR2* transcription was determined after another 1 h culture. The expression level of *FgQDR2* in the non-treated strain was referred to as 1, and the *FgACTIN* gene was used as the internal control for normalization. (**E**) Analysis of the phosphorylation sites within the LOOP substrate binding region of Qdr2. The phosphorylation site of FgQdr2 was predicted using NetPhos 3.1 Server. The LOOP region of FgQdr2 was predicted using the Swiss-Model Repository program. For (**C**,**D**), mean and standard deviation were estimated with data from three independent biological replicates (*n* = 3). Different letters indicate significant differences based on ANOVA analysis, followed by Turkey’s multiple comparisons test (*p* < 0.05).

To elucidate the underlying mechanism of FgQdr2 in multiple drug resistance, the subcellular location of FgQdr2 was examined. Unfortunately, we failed to observe the GFP signals after scanning more than 100 complemented strains, although the FgQdr2-GFP protein could be detected by Western blot assays (Figure 2A and Figure S4A). We then attempted to increase the expression level of *FgQDR2* by replacing the native promoter of *FgQDR2* with the *gpdA* promoter from *A. nidulans*. The overexpressed strains (ΔFgQdr2::Qdr2^OE^-GFP) were verified by quantitative reverse transcription PCR (qRT-PCR) assays (Figure S4). The confocal microscope examination of the ΔFgQdr2::Qdr2^OE^-GFP strain showed that the overexpressed FgQdr2 is localized along the plasma membrane (Figure 2A), which corresponds to its role as the membrane transporter in transporting solutes upon the chemiosmotic ion gradients. Surprisingly, phenotypic analysis of drug sensitivity in the ΔFgQdr2::Qdr2^OE^-GFP strain found that overexpression of FgQdr2 did not improve the drug resistance to prochloraz, tebuconazole, fludioxonil, or iprodione (Figure 2B,C). Accordingly, the transcription level of *FgQDR2* was not induced by the treatment with the fungicides prochloraz, tebuconazole, fludioxonil, or iprodione, according to qRT-PCR assays (Figure 2D). Thus, it is deduced that the expression of DHA1 transporter FgQdr2 under normal growth conditions is comparatively low, but sufficient for the regulation of drug efflux in *F. graminearum*.

### 3.4. The Predicted Phosphorylation Sites and LOOP Region within FgQdr2 Are Not Required for Its Function in Drug Resistance

In eukaryotic cells, the functions of approximately one-third of proteins are regulated via phosphorylation/dephosphorylation [43], and phosphorylation occurs mainly on three hydroxyl-containing amino acids—serine (Ser), threonine (Thr) and tyrosine (Tyr)—with Ser as the predominant target [44]. The analysis of the phosphoproteome profiling assay in our previous study, combined with the protein phosphorylation site prediction program NetPhos 3.1 (http://www.cbs.dtu.dk/services/NetPhos/ (20 September 2022)), found that FgQdr2 was assumed to be phosphorylated at S260, T264, T282, and S288 (Figure 2E). According to the FgQdr2 tertiary structure prediction using the Swiss-Model Repository program (https://swissmodel.expasy.org/ (20 September 2022)), these phosphorylation sites are localized in the LOOP region (259-313 aa, Figure 2E), which is essential for MSF transmitting conformational changes during substrate transport [45,46], indicating the potential regulation roles of FgQdr2 phosphorylation in substrate transport and drug resistance. To further verify this hypothesis, we constructed a strain expressing constitutive dephosphorylated FgQdr2 by replacing, point to point, the four predicted phosphorylated S/T residues with the alanine (A), designated as ΔFgQdr2-C^S260A/T264A/T282A/S288A^. However, ΔFgQdr2-C^S260A/T264A/T282A/S288A^ showed similar drug sensitivity to ΔFgQdr2-C and PH-1 (Figure 2B,C), suggesting that the four phosphorylation sites of FgQdr2 are not associated with drug extrusion in *F. graminearum*. Additionally, we also constructed the LOOP region deletion mutant ΔFgQdr2-C^ΔLOOP^; phenotypic characterization found that the strain lacking the LOOP region showed no significant difference in drug sensitivity compared with ΔFgQdr2-C and PH-1 (Figure 2B,C). Taken together, these results indicated that the LOOP region, as well as the predicted phosphorylation sites within it, are not necessary for the function of FgQdr2 in drug resistance. The specific mechanism of FgQdr2 involved in multiple drug resistance requires further investigation.

### 3.5. FgQdr2 Is Involved in Potassium, Sodium, and Copper Transport, as Well as Membrane Proton Gradient Regulation

**Figure 3:**
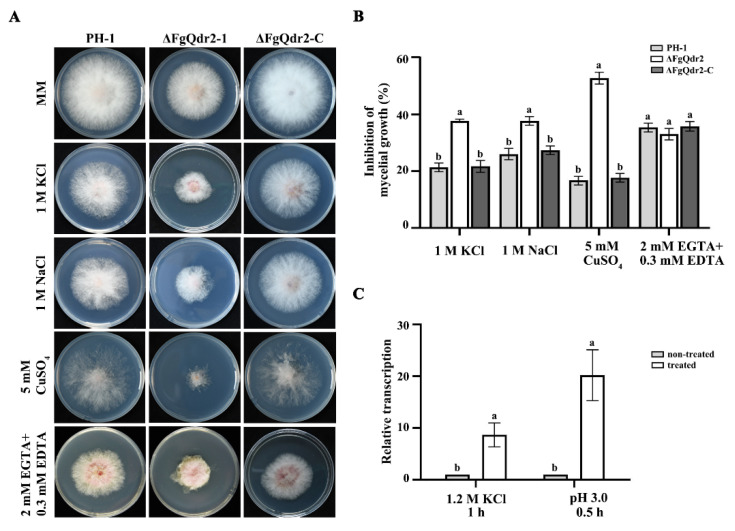
FgQdr2 is involved in the transport of potassium, sodium, and copper, as well as the regulation of membrane proton gradient. (**A**) Sensitivity of each strain to the corresponding stress agents. ΔFgQdr2 exhibited increased sensitivity to K^+^ Na^+^, Cu^2+^ osmotic stresses, and displayed similar sensitivity to the divalent cation chelator ethylene diamine tetra-acetic acid (EDTA), compared with the wild type. Each strain was cultured on MM, with or without the corresponding stress agents. The images were taken after 3 d incubation at 25 °C, and the mycelial growth inhibition was calculated for each strain. (**B**) The mycelial growth inhibition of each strain under the above drug treatments. (**C**) The qRT-PCR revealed that the transcription level of *FgQDR2* was increased under K^+^ and H^+^ inductions. The stain was first cultured in yeast extract peptone dextrose (YEPD) for 24 h, then transferred to YEPD supplied with (treated) or without (non-treated) the corresponding ion stress agents. The expression level of *FgQDR2* in the non-treated strain was referred to as 1, and the *FgACTIN* gene was used as the internal control for normalization. For (**B**,**C**), mean and standard deviation were estimated with data from three independent biological replicates (*n* = 3). Different letters indicate significant differences based on ANOVA analysis, followed by Turkey’s multiple comparisons test (*p* < 0.05).

Besides its roles in multiple drug efflux, Qdr2 has also been connected to potassium transport in yeast [47]. To assess whether the efflux of potassium was dependent on the FgQdr2 in *F. graminearum*, an assessment of ΔFgQdr2 sensitivity towards potassium-involved osmotic stress was conducted. As seen in Figure 3A,B, ΔFgQdr2 showed increased sensitivity towards osmotic stress (1M KCl). Consistently, qRT-PCR assays showed that the expression of *FgQDR2* was massively increased under osmotic stress (treated with 1.2 M KCl, Figure 3C). Additionally, the expression of *FgQDR2* was also upregulated in the presence of massive H^+^ (pH 3.0, Figure 3C), indicating its predicted role in membrane proton gradient regulation during substrate transport. Further investigation of the spectrum of cations transported by FgQdr2 found that FgQdr2 was related to transition metals, such as sodium and copper (Figure 3A,B). To determine whether FgQdr2 is involved in the transport of the divalent cation, as reported in yeast [36], we treated ΔFgQdr2 with the divalent cation (calcium or magnesium) chelators—ethylene diamine tetra-acetic acid (EDTA) and ethylene glycol tetra-acetic acid (EGTA)—and found that ΔFgQdr2 displayed similar sensitivity to the wild type (Figure 3A,B), suggesting that FgQdr2 may be not involve in calcium or magnesium extrusion in *F. graminearum*. Taken together, these results indicated that FgQdr2 acts as a key factor in osmotic adjustment by balancing potassium homeostasis, and it is also involved in sodium, as well as copper, transport, which may regulate substrate efflux in a proton gradient-dependent manner.

### 3.6. FgQdr2 Is Required for the Virulence of F. graminearum

**Figure 4:**
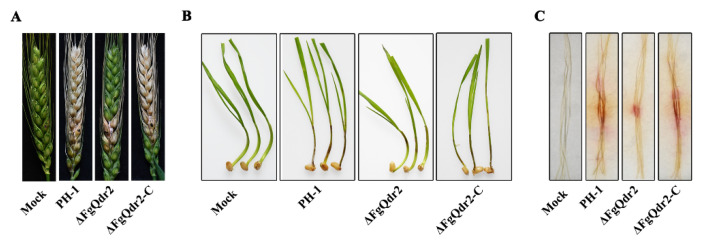
FgQdr2 is important for the virulence of *F. graminearum*. Virulence of each strain on wheat heads (**A**), coleoptiles (**B**), and corn silks (**C**). The mutant ΔFgQdr2 showed significantly reduced virulence on the above wheat tissues. The mock treatments indicate the corresponding host tissues inoculated with sterile water as a negative control.

Considering the significant roles of FgQdr2 in facilitating ions and solutes moving through the membranes, we speculated that FgQdr2, besides regulating growth, may be crucial for the infection of *F. graminearum*. As expected, scab symptoms caused by ΔFgQdr2 were restricted to the inoculated spikelet and the adjacent two or three spikelets fifteen days after inoculation (Figure 4A), whereas the wild type and the complemented strains caused typical scab symptoms on wheat heads under the same condition (Figure 4A). Additionally, the examination of virulence on wheat coleoptiles showed that ΔFgQdr2 infected coleoptiles displayed shorter brown necrotic lesions than those of PH-1 and ΔFgQdr2-C infected coleoptiles (Figure 4B), indicating the reduced virulence of ΔFgQdr2 on wheat coleoptiles. Accordingly, virulence assay on corn silks showed that ΔFgQdr2 also demonstrated attenuated virulence on corn silks (Figure 4C), suggesting that FgQdr2 is associated with the virulence of *F. graminearum*. Taken together, these results indicated that FgQdr2 plays important roles in the pathogenesis of *F. graminearum*.

### 3.7. FgQdr2 Is Important for DON Production and Phytoalexin Extrusion during F. graminearum Infection

**Figure 5:**
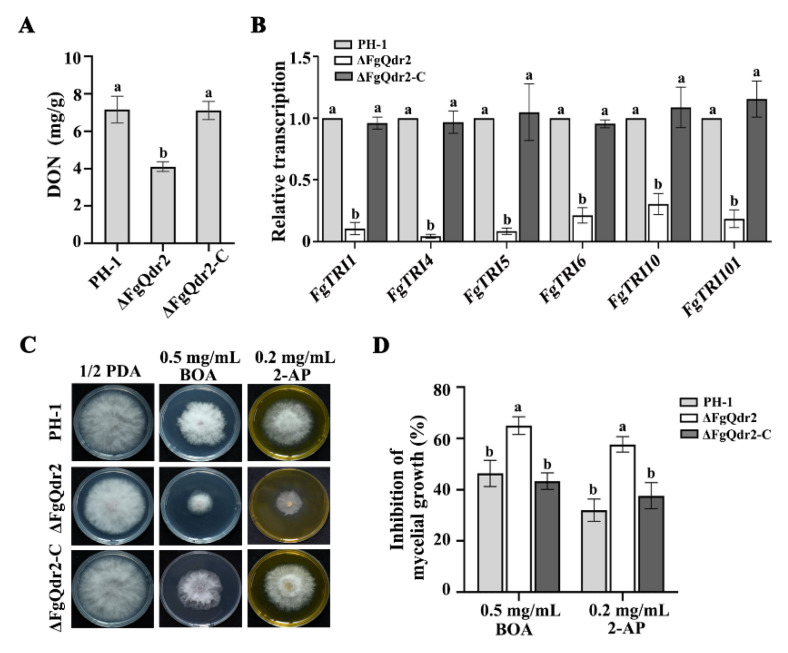
FgQdr2 is required for DON production and phytoalexin extrusion in *F. graminearum*. (**A**) DON content determination of each strain. ΔFgQdr2 showed significant deficiency in DON biosynthesis. (**B**) The gene expression level of *FgTRI* genes in each strain. The expression level of each *FgTRI* in PH-1 in TBI with putrescine was referred to as 1, and the *FgACTIN* gene was used as the internal control for normalization. (**C**) Sensitivity of each strain to phytoalexin BOA and 2-AP. ΔFgQdr2 showed increased sensitivity towards the two phytoalexins shown above. (**D**) Hyphal growth inhibition rate under with the above phytoalexins. In (**A**–**C**), mean and standard deviation were estimated with data from three independent biological replicates (*n* = 3). Different letters indicate significant differences based on ANOVA analysis, followed by Turkey’s multiple comparisons test (*p* < 0.05).

Given that DON is proposed as an important virulence factor in *F. graminearum* [4,5,6,7,8], and ΔFgQdr2 showed a significant defect in virulence, we then assayed DON biosynthesis in ΔFgQdr2. After cultivation in the TBI medium for 7 d, ΔFgQdr2 was found to produce significantly lower levels of DON than the PH-1 and its complemented strains (Figure 5A). To further confirm the results, the transcription levels of the core DON biosynthetic genes (*FgTRI*s) [48] was determined using qRT-PCR assays. Accordingly, the expression levels of *FgTRI1*, *FgTRI4*, *FgTRI5*, *FgTRI6*, *FgTRI10*, and *FgTRI101* in ΔFgQdr2 were dramatically decreased by 89.4%, 95.7%, 91.6%, 78.7%, 69.5%, and 81.5%, respectively, as compared to those in the PH-1 and its complemented strains (Figure 5B). These results suggested that FgQdr2 is involved in the biosynthesis of the virulence factor DON during disease spreading.

The major phytoalexins produced by wheat during the defense response to various biotic stresses are 2-benzoxazolinone (BOA) and its degradation product 2-AP (2-aminophenoxazin-3-one) [49]. Previous studies found that detoxifying the benzoxazolinone class of phytoalexins is important for the virulence of *Fusarium* [50]. To further illustrate the function of FgQdr2 during the infection, we therefore detected the sensitivity changes to BOA and 2-AP upon the deletion of *FgQDR2*. Phenotypic characterization showed that ΔFgQdr2 was hypersensitive to BOA and 2-AP (Figure 5C,D), indicating that FgQdr2 may be involved in BOA and 2-AP detoxification during infection. Taken together, these results suggested that the defects in DON production and the scavenging of the phytoalexin contributes to the reduced virulence of the ΔFgQdr2 mutant. 

### 3.8. FgQdr2 Is Essential for Asexual Sexual Development in F. graminearum

**Figure 6:**
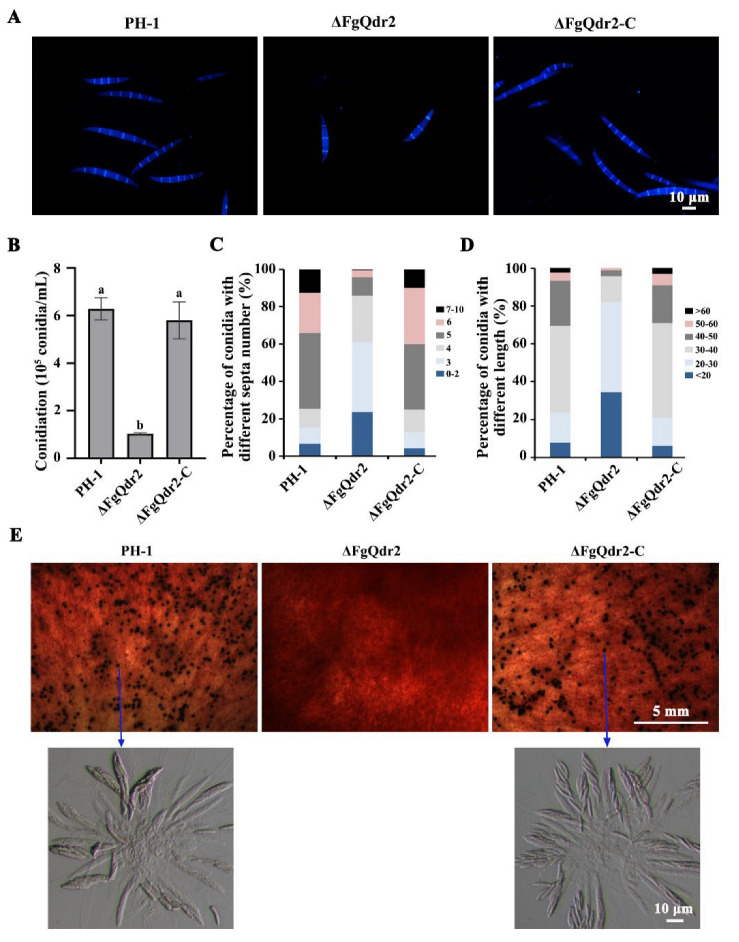
The deletion of *FgQDR2* resulted in defects in sexual and asexual reproduction in *F. graminearum*. (**A**) The spores of the wild-type and ΔFgQdr2 were stained with calcium fluorescent white (CFW). Bar = 10 μm. (**B**) The number of conidia produced by each strain. (**C**) The number of spore septa in each strain. A total of 200 conidia were examined for each strain. (**D**) The spore length in each strain. A total of 200 conidia were examined for each strain. (**E**) Deletion of *FgQDR2* leads to sexual reproduction defects in *F. graminearum*.

Asexual and sexual reproduction plays a critical role in the infection cycle of homothallic fungus *F. graminearum* [38]. To further investigate the roles of FgQdr2 during the FHB disease process, we characterized its roles in asexual and sexual development. As showed in Figure 6A–D, ΔFgQdr2 exhibited decreased conidiation in CMC liquid medium, as well as shortened conidia, with fewer septa. Additionally, the examination of perithecium formation on carrot agar mating cultures showed that the ΔFgQdr2 was sterile (Figure 6E), whereas the wild type and complemented strains produced mature perithecia containing normal asci (Figure 6E). These results suggested that FgQdr2 is important for asexual and sexual reproduction in *F. graminearum* during the FHB disease cycle.

## 4. Discussion

FHB caused by *F. graminearum* is one of the most devastating diseases on wheat, barley, maize, and other important cereal crops worldwide [1,2]. The management of this disease is becoming increasingly difficult due to the limited number of effective antifungal drugs against DON pollution and fungi spreading, as well as the emergence of drug resistance [16,17,18,19,20]. Drug resistance is often attributed to the action of multidrug efflux pumps belonging to the ABC and MFS families [22,51]. However, while many studies have focused on the role of ABC multidrug efflux transporters, little is yet known regarding the part played by the drug H^+^ antiporter (DHA) family of the MFS [25,27]. In this study, a systematic gene deletion analysis of all putative DHA transporter genes in *F. graminearum* was carried out. Surprisingly, among the 34 DHA transporters, only FgQdr2 exhibited severe deficiency in multiple drug resistance and growth (Figure 1A,B, Table S2). In *S. cerevisiae*, ScQdr2 has been reported to confer tolerance to multiple drugs, including the antimalarial drug quinidine, the herbicide barban, and the anti-cancer agents cisplatin and bleomycin [37,52]. Accordingly, *C. glabrata* Qdr2 (CgQdr2), as the first DHA reported in pathogenic yeast, was also identified as playing important roles in the resistance of antifungal drugs, including quinidine, miconazole, tioconazole, clotrimazole, and ketoconazole. Notably, neither ScQdr2 nor CgQdr2 confer resistance to triazoles [27]. Interestingly, our phenotypic characterization found that FgQdr2 is involved in the efflux of multiple fungicides (including fludioxonil and iprodione), as well as triazoles (prochloraz and tebuconazole), indicating the slight difference among *F. graminearum*, *S. cerevisiae*, and *C. glabrata* in the spectrum of antifungal drugs transported by Qdr2 (Figure 1A,B).

Although Qdr2 has been characterized as the efflux pump of a wide variety of drugs, the molecular mechanisms behind its functions remain elusive and controversial [22,23,27]. The study of *C. glabrata* found that CgQdr2 functioning as a clotrimazole efflux was localized to the plasma membrane as ScQdr2, and its transcript level was significantly increased (3-fold) upon the induction of clotrimazole [27], indicating that the overexpression of CgQdr2 is one strategy for *C. glabrata* to confer chemical stress. In *F. graminearum*, the confocal microscope examination of the ΔFgQdr2::Qdr2^OE^-GFP strain showed that the FgQdr2 was localized along the plasma membrane (Figure 2A), indicating that it has the same subcellular location as that of *C. glabrata* and *S. cerevisiae* [27,36]. However, qRT-PCR assays showed that the treatment with FgQdr2-exported fungicides did not induce the transcription of *FgQDR2* (Figure 2D). Accordingly, the overexpression of FgQdr2 did not improve the drug resistance to FgQdr2-exported fungicides (Figure 2B,C), indicating the different mechanism associated with FgQdr2 against chemical stress in *F. graminearum*. Considering that the functions of approximately one-third of proteins are regulated via phosphorylation/dephosphorylation in eukaryotes [43], we then performed the phosphorylation site prediction for FgQdr2 to further illustrate its mechanism associated with drug extrusion (Figure S5). However, neither the predicted phosphorylation sites, nor the LOOP region responsible for substrate recognition, were necessary for the function of FgQdr2 in drug resistance (Figure 2B,C). Notably, different from drug treatments, the expression of *FgQDR2* was upregulated in the presence of massive H^+^ (Figure 3C), indicating that it may be associate with the maintenance of the proton gradient across the plasma membrane during substrate transport. Thus, we speculated that the transcription of FgQdr2 may first be activated in response to the changes of proton gradient, then proceed due to the environmental chemical stresses. However. the specific mechanism of FgQdr2 involved in drug resistance requires further investigation.

Besides working as drugs efflux pumps, ScQdr2 also plays a prominent role in maintaining ion homeostasis to provide the first layer of sophisticated regulation at the boundary between an organism and its environment. In *S. cerevisiae*, ScQdr2 was proposed to have a role in potassium homeostasis [47], while CgQdr2 is not involved in potassium transport [27]. Additionally, phenotypic characters of *S. cerevisiae* showed that ScQdr2 is associated with the transport of sodium, manganese, calcium, and copper [36]. However, in *F. graminearum*, FgQdr2 acts as a key factor in osmotic adjustment by balancing potassium homeostasis, and it is also involved in sodium and copper transport, but not in divalent cations (Figure 3A,B), suggesting a slight difference in the physiological substrate of Qdr2 among species.

Owing to its significance in aiding the movement of ions and solutes through the membranes via facilitating diffusion, symport, or antiport, MFS is crucial for multiple physiological processes including cell growth, reproduction, and even virulence in pathogenetic fungi [29,53]. For example, CaQdr2 is responsible for the virulence and biofilm formation in *C. albicans* [54]. Consistently, deletion of FgQdr2 also resulted in attenuated pathogenicity in both wheat and corn (Figure 4A–C). Moreover, FgQdr2 was found to be associated with fungal infection via regulating the biosynthesis of virulence factor DON (Figure 5A,B), the scavenging of the phytoalexin BOA and PAO (Figure 5C,D), as well as both the asexual and sexual reproduction in *F. graminearum* (Figure 6). Overall, our study demonstrated the functions of FgQdr2 in drug resistance, growth, and balancing ion homeostasis and virulence; however, the specific mechanism of FgQdr2 involved in drug resistance and the puzzle regarding whether the physiological role of FgQdr2 in ion transport contributes indirectly to its functions in multidrug resistance and pathogenicity still require further exploration.

## Data Availability

The data in this study are available in this article.

## Supplementary Materials

The following supporting information can be downloaded at: https://www.mdpi.com/article/10.3390/jof8101009/s1, Table S1. A list of PCR primers used in this study and their relevant characteristics; Table S2: The collection of phenotypic analyses for 34 DHA transporter deletion mutants of *F. graminearum*.
