# Supplementary material for: The Drug H+ Antiporter FgQdr2 Is Essential for Multiple Drug Resistance, Ion Homeostasis, and Pathogenicity in Fusarium graminearum
Source: J Fungi (Basel). 2022 Sep 26;8(10):1009. doi: 10.3390/jof8101009 (PMC9605015; doi:10.3390/jof8101009)
Supplement: Supplementary file 1 [file jof-08-01009-s001.zip › jof-1934311-Supplementary Files-latext/Figures.pdf]

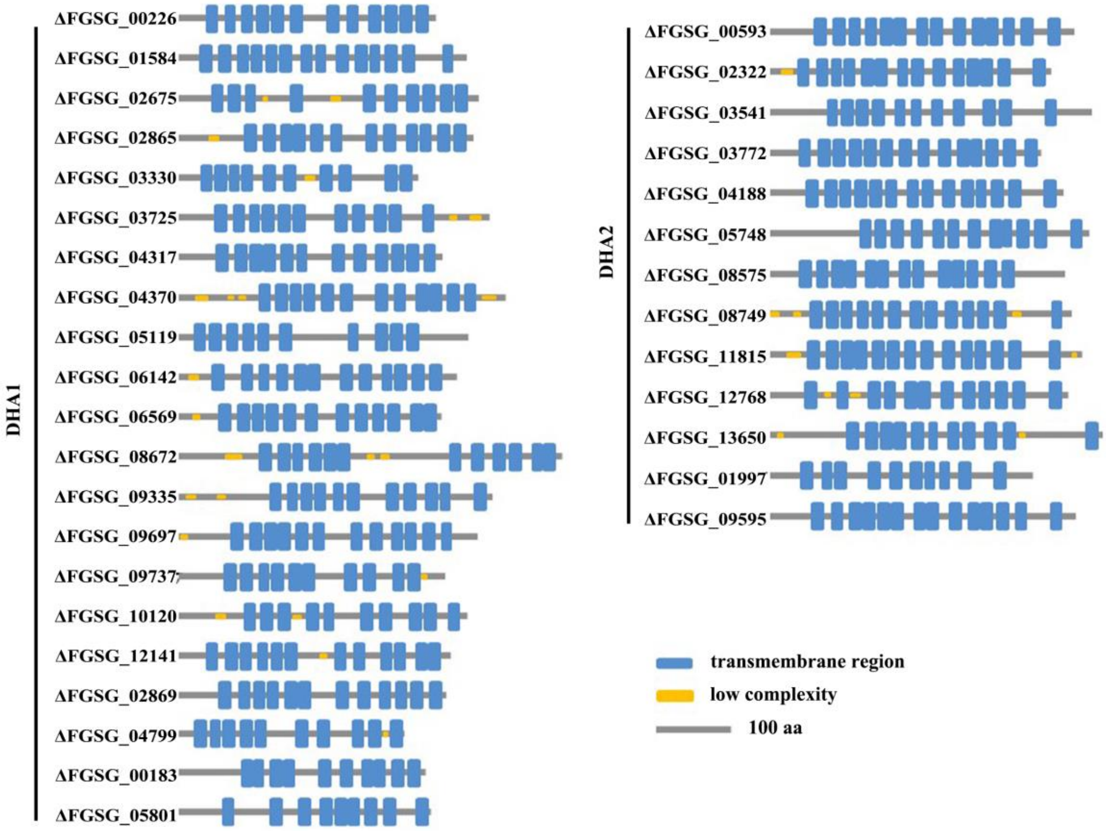

Supplementary Figure S1. Domain structures of 34 putative H+ antiporters (DHAs) in *F. graminearum*. The domains of 34 putative DHA transporters that are distributed two subfamilies DHA1 and DHA2 were analyzed by SMART protein database (http://smart.emblheidelberg.de).

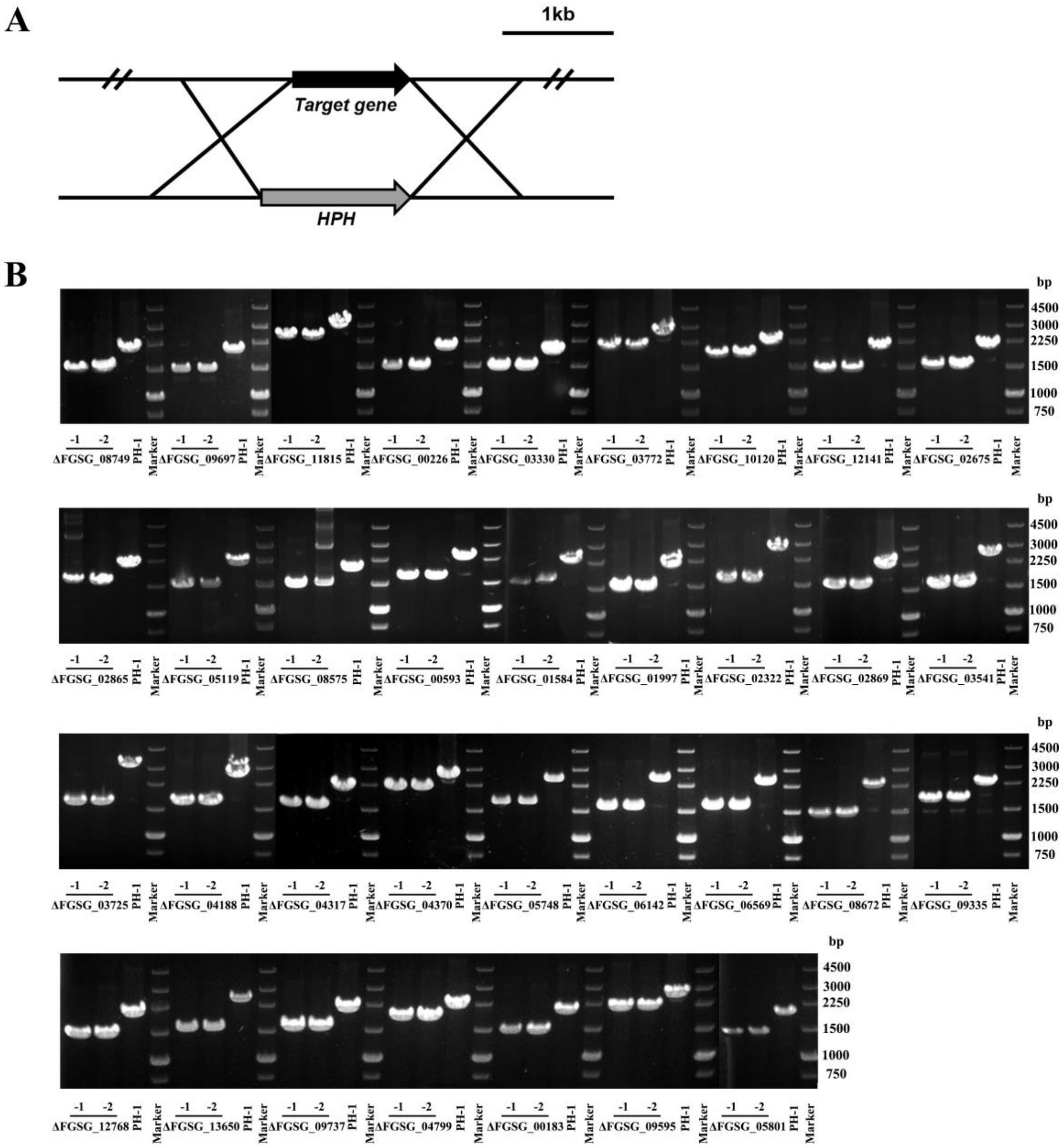

Supplementary Figure S2. Schematic diagram of gene disruption strategy and PCR identification for 34 DHA genes. (**A**) In the schematic diagram of gene disruption strategy, gray arrow indicates the resistant fragment hygromycin (*HPH*) used for replacement of the target gene, and the black arrow represents the target gene to be knocked out. (**B**) PCR identification of 34 gene deletion mutants of DHA transporters.

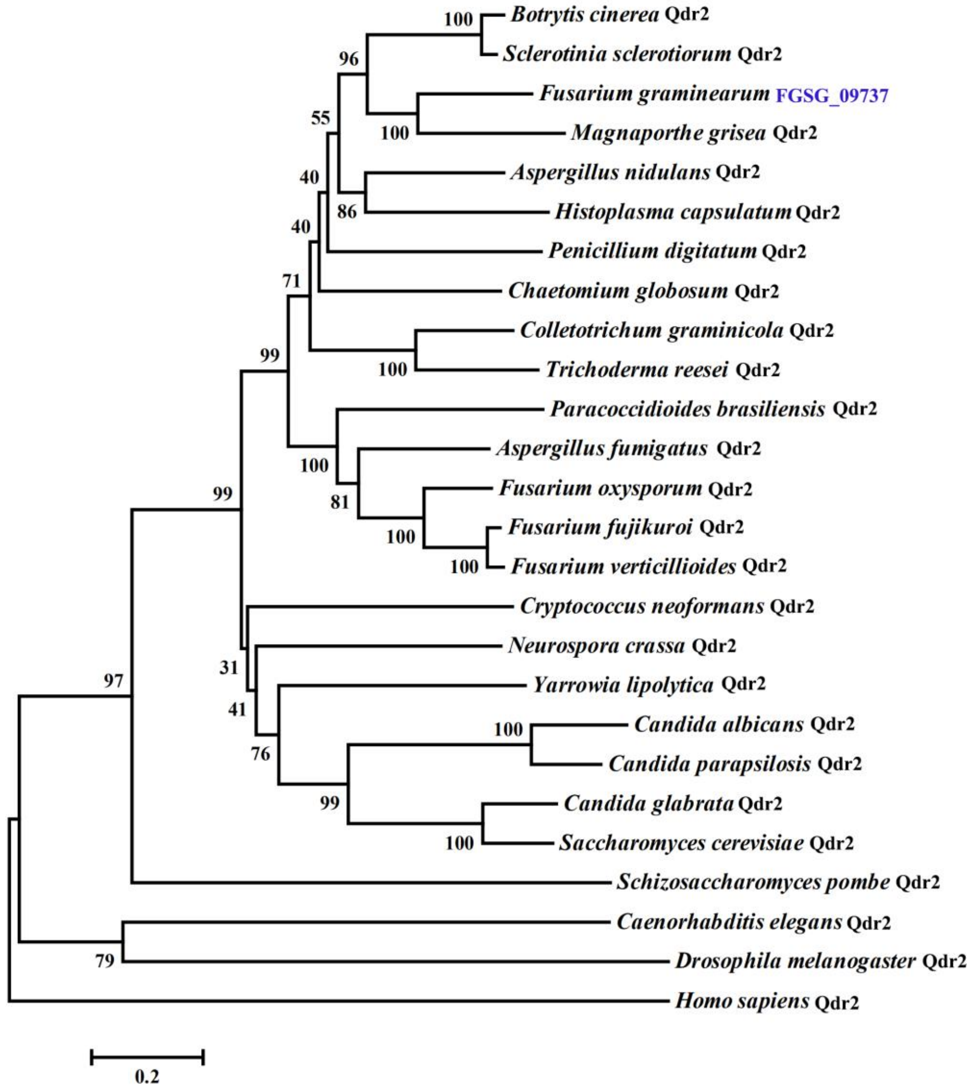

Supplementary Figure S3. Phylgenetic tree of FgQdr2 and its orthologs in other organisms. The homology among Qdr2 orthologs from 26 eukaryotic species was analyzed using the Protein BLAST tool on the NCBI platform (https://blast.ncbi.nlm.nih.gov/Blast.cgi). The phylogenetic tree was constructed based on the Qdr2 amino acid sequences from 26 species with Mega 5.0 using the neighborjoining method. The number at the branches indicate the bootstrap values from 1000 replications.

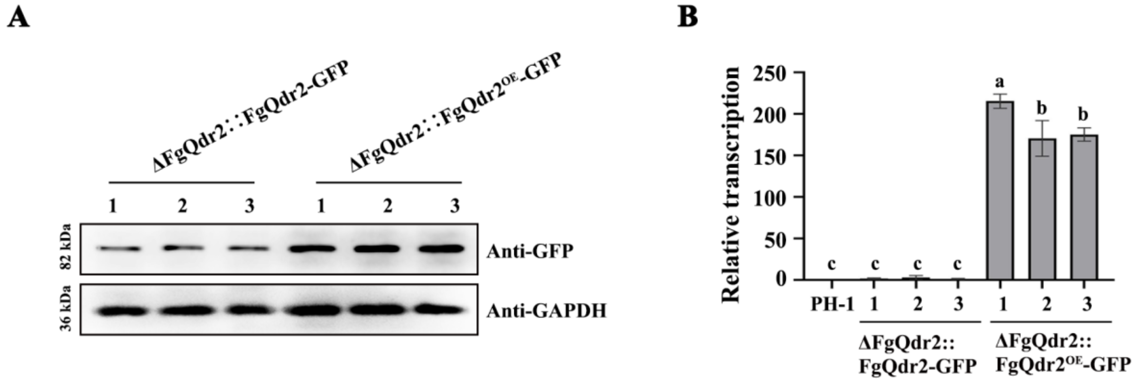

Supplementary Figure S4. Construction and identification of FgQdr2 complemented and overexpressed strains. (**A**) Western blot of ΔFgQdr2::Qdr2-GFP and ΔFgQdr2::Qdr2OE-GFP strains. GAPDH level was used as the protein loading reference. (**B**) Relative transcription levels of *FgQDR2* in ΔFgQdr2::Qdr2OE-GFP strains in YEPD. The expression level of *FgQDR2* in PH-1 was referred to 1 and the FgACTIN gene was used as the internal control for normalization. Mean and standard deviation were estimated with data from three independent biological replicates (n = 3). Different letters indicate significant differences based on ANOVA analysis followed by Turkey's multiple comparisons test ($p < 0.05$).
