# Supplementary material for: The Drug H+ Antiporter FgQdr2 Is Essential for Multiple Drug Resistance, Ion Homeostasis, and Pathogenicity in Fusarium graminearum
Source: J Fungi (Basel). 2022 Sep 26;8(10):1009. doi: 10.3390/jof8101009 (PMC9605015; doi:10.3390/jof8101009)
Supplement: Supplementary file 1 [file jof-08-01009-s001.zip › jof-1934311-Supplementary Files-latext/Table S1.pdf]

**Table S1**. A list of PCR primers used in this study and their relevant characteristics.		
Primer	Sequence(5’-3’)	Relevant Characteristics
00593-UP-F	TTGGGAGACTGGGTTGAA	PCR primers for amplification of the upstream fragment of 00593
00593-UP-R	CAAAATAGGCATTGATGTGTTGACCTCCAGAACAAACGACGGGAAT	PCR primers for amplification of the upstream fragment of 00593
00593-DOWN-F	CTCGTCCGAGGGCAAAGGAATAGAGTAGCTGAAGCATTCCAAATAACAC	PCR primers for amplification of the downstream fragment of 00593
00593-DOWN-R	CTGACTTTTGAGCCTGTTT	PCR primers for amplification of the downstream fragment of 00593
00593-ID-2545-F	TTACCCCAGTTGATTTGATTG	PCR primers for the identification of 00593 disruption
00593-ID-1738-R	TCGGCTGAGGAGCGGATT	PCR primers for the identification of 00593 disruption
01584-UP-F	CTCAACATTCCTGGGTCT	PCR primers for amplification of the upstream fragment of 01584
01584-UP-R	CAAAATAGGCATTGATGTGTTGACCTCCAAATCATCTGGTCCTTGC	PCR primers for amplification of the upstream fragment of 01584
01584-DOWN-F	CTCGTCCGAGGGCAAAGGAATAGAGTAGCCAAGACTGACCATTCGC	PCR primers for amplification of the downstream fragment of 01584
01584-DOWN-R	TTTTCTCACGCCCAACAC	PCR primers for amplification of the downstream fragment of 01584
01584-ID-2309-F	GGGAGGGTGAACGATGTA	PCR primers for the identification of 01584 disruption
01584-ID-1553-R	CGGTCCTTTGTGGTGAGA	PCR primers for the identification of 01584 disruption
01997-UP-F	AGCCATTTTCTTGTCAGTC	PCR primers for amplification of the upstream fragment of 01997
01997-UP-R	CAAAATAGGCATTGATGTGTTGACCTCCGTTTAGCCCTACCTTGATG	PCR primers for amplification of the upstream fragment of 01997
01997-DOWN-F	CTCGTCCGAGGGCAAAGGAATAGAGTAGTTACTGCCAAAGTGACGC	PCR primers for amplification of the downstream fragment of 01997
01997-DOWN-R	TTCCAAGACTCCGATGTG	PCR primers for amplification of the downstream fragment of 01997
01997-ID-2617-F	GGTTCTCGTCGTATTTGC	PCR primers for the identification of 01997 disruption
01997-ID-1600-R	GTGTTTGAGGCGGTAGAT	PCR primers for the identification of 01997 disruption
02055-UP-F	GGCACGGACAACAAGCAG	PCR primers for amplification of the upstream fragment of 02055
02055-UP-R	CAAAATAGGCATTGATGTGTTGACCTCCCAGCCGAACGATGGTGAT	PCR primers for amplification of the upstream fragment of 02055

02055-DOWN-F	CTCGTCCGAGGGCAAAGGAATAGAGTAGGTAAGTCCCAGAGTCTCAATG	PCR primers for amplification of the downstream fragment of 02055
02055-DOWN-R	GGAAGAGCACCAAAAGTCA	PCR primers for amplification of the downstream fragment of 02055
02055-ID-3048-F	TCTCAATAAGGAACCACGAA	PCR primers for the identification of 02055 disruption
02055-ID-1609-R	GCCTACGGTCAATACATCA	PCR primers for the identification of 02055 disruption
02322-UP-F	GGATGGACATTGCTGATAA	PCR primers for amplification of the upstream fragment of 02322
02322-UP-R	CAAAATAGGCATTGATGTGTTGACCTCCAAAACTGACCTTCGGGAC	PCR primers for amplification of the upstream fragment of 02322
02322-DOWN-F	CTCGTCCGAGGGCAAAGGAATAGAGTAGTCTATCACAGAAGGCACTAAT	PCR primers for amplification of the downstream fragment of 02322
02322-DOWN-R	AAGTTCACGAGCGACACC	PCR primers for amplification of the downstream fragment of 02322
02322-ID-2502-F	CATCAGTAAGCGGACAGG	PCR primers for the identification of 02322 disruption
02322-ID-1641-R	TATTAGGGCATCCGTGTT	PCR primers for the identification of 02322 disruption
02869-UP-F	ATTCGGGGCTACTTTCAG	PCR primers for amplification of the upstream fragment of 02869
02869-UP-R	CAAAATAGGCATTGATGTGTTGACCTCCGCTAACCGTTCATTACCTCT	PCR primers for amplification of the upstream fragment of 02869
02869-DOWN-F	CTCGTCCGAGGGCAAAGGAATAGAGTAGGTTGGGAGTTTGTGGTAGA	PCR primers for amplification of the downstream fragment of 02869
02869-DOWN-R	GTTCTGAAGTGAGGGCTAA	PCR primers for amplification of the downstream fragment of 02869
02869-ID-2026-F	TGGCTGTTTTGCTTTCTT	PCR primers for the identification of 02869 disruption
02869-ID-1515-R	CTACCCAGTCGTGTATTGC	PCR primers for the identification of 02869 disruption
03541-UP-F	GCAAAATACTGGGCTGTC	PCR primers for amplification of the upstream fragment of 03541
03541-UP-R	CAAAATAGGCATTGATGTGTTGACCTCCGGGTGAACTTGACTTGGA	PCR primers for amplification of the upstream fragment of 03541
03541-DOWN-F	CTCGTCCGAGGGCAAAGGAATAGAGTAGGCTTGCTTATTGCCTCCT	PCR primers for amplification of the downstream fragment of 03541
03541-DOWN-R	CAGCCTCACCAGACTTGC	PCR primers for amplification of the downstream fragment of 03541
03541-ID-2474-F	TGTCTCCCTTATCTCACCTT	PCR primers for the identification of 03541 disruption
03541-ID-1718-R	CCTCGTTCCAACTTTTACT	PCR primers for the identification of 03541 disruption
03725-UP-F	CATCAAAGTCTGGAGCATAA	PCR primers for amplification of the upstream fragment of 03725
03725-UP-R	CAAAATAGGCATTGATGTGTTGACCTCCGGGACGGATTTGTAGGTAG	PCR primers for amplification of the upstream fragment of 03725

03725-DOWN-F	CTCGTCCGAGGGCAAAGGAATAGAGTAGTCCCTGTTGAACGAGTGC	PCR primers for amplification of the downstream fragment of 03725
03725-DOWN-R	CGTATGTAGCGATAAGCAGTT	PCR primers for amplification of the downstream fragment of 03725
03725-ID-2492-F	CTGTCCAGGGTGTTGTCG	PCR primers for the identification of 03725 disruption
03725-ID-1672-R	TGGGCAGTGGGCTATGTT	PCR primers for the identification of 03725 disruption
04188-UP-F	AGTCGTGATAAGCGTTGAG	PCR primers for amplification of the upstream fragment of 04188
04188-UP-R	CAAAATAGGCATTGATGTGTTGACCTCCGGGTGTAGTGTATCGGTGT	PCR primers for amplification of the upstream fragment of 04188
04188-DOWN-F	CTCGTCCGAGGGCAAAGGAATAGAGTAGGGGGTTCGTTGTGAGTGG	PCR primers for amplification of the downstream fragment of 04188
04188-DOWN-R	AGGCGGGAGGAAGTTGAG	PCR primers for amplification of the downstream fragment of 04188
04188-ID-2224-F	CTATGCTGCGGTTTTGTG	PCR primers for the identification of 04188 disruption
04188-ID-1546-R	CAGACGCCGTTCTATGTG	PCR primers for the identification of 04188 disruption
04317-UP-F	CTGGTTGGTCCGTAGTGC	PCR primers for amplification of the upstream fragment of 04317
04317-UP-R	CAAAATAGGCATTGATGTGTTGACCTCCTGAAAGGATGCCTGTTGC	PCR primers for amplification of the upstream fragment of 04317
04317-DOWN-F	CTCGTCCGAGGGCAAAGGAATAGAGTAGGAAAGCCTGACAGTAAAATG	PCR primers for amplification of the downstream fragment of 04317
04317-DOWN-R	GAAAGGAAATACTACCAACG	PCR primers for amplification of the downstream fragment of 04317
04317-ID-2263-F	AACTTACCGTTACCTCCCC	PCR primers for the identification of 04317 disruption
04317-ID-1707-R	GCGTCCGACTTGCTAAAA	PCR primers for the identification of 04317 disruption
04370-UP-F	TAATCTACGGGAATGGGA	PCR primers for amplification of the upstream fragment of 04370
04370-UP-R	CAAAATAGGCATTGATGTGTTGACCTCCGAAACAGGAAAAGGGTCA	PCR primers for amplification of the upstream fragment of 04370
04370-DOWN-F	CTCGTCCGAGGGCAAAGGAATAGAGTAGGAGACGGCTATGGAACGC	PCR primers for amplification of the downstream fragment of 04370
04370-DOWN-R	GGATGAACTCGGCTGGTG	PCR primers for amplification of the downstream fragment of 04370
04370-ID-2832-F	TTTTACCAGGTTCGTCTTG	PCR primers for the identification of 04370 disruption
04370-ID-1678-R	GCATCCTTTGTCCTTTCA	PCR primers for the identification of 04370 disruption
05748-UP-F	CCTAAACGGTCTAATCTGG	PCR primers for amplification of the upstream fragment of 05748
05748-UP-R	CAAAATAGGCATTGATGTGTTGACCTCCCGCATCCTCTGAGCAAGT	PCR primers for amplification of the upstream fragment of 05748

05748-DOWN-F	CTCGTCCGAGGGCAAAGGAATAGAGTAGGTAATGAGTATGGTTGGGTG	PCR primers for amplification of the downstream fragment of 05748
05748-DOWN-R	CGGTATGATTCCTGCTTT	PCR primers for amplification of the downstream fragment of 05748
05748-ID-2389-F	CCGTTGTAAGACGAAAAGT	PCR primers for the identification of 05748 disruption
05748-ID-1545-R	CTGACGATGTTAGAGGAGG	PCR primers for the identification of 05748 disruption
06142-UP-F	AGCGAAGTGAAGGGGAGT	PCR primers for amplification of the upstream fragment of 06142
06142-UP-R	CAAAATAGGCATTGATGTGTTGACCTCCCAAGATAATCAAGGGAGGAAA	PCR primers for amplification of the upstream fragment of 06142
06142-DOWN-F	CTCGTCCGAGGGCAAAGGAATAGAGTAGTCCAAATACCCACCCAAAT	PCR primers for amplification of the downstream fragment of 06142
06142-DOWN-R	CAGTCAAGGCAAAAGAATC	PCR primers for amplification of the downstream fragment of 06142
06142-ID-2382-F	TAGCGGGTAGCAGAAAAG	PCR primers for the identification of 06142 disruption
06142-ID-1844-R	GGAGAACCTGGAGACGAA	PCR primers for the identification of 06142 disruption
06569-UP-691-F	GCCTGTTTTATCTAATGCTAC	PCR primers for amplification of the upstream fragment of 06569
06569-UP-R	CAAAATAGGCATTGATGTGTTGACCTCCTGGATGTGCGACTTTACTA	PCR primers for amplification of the upstream fragment of 06569
06569-DOWN-684-F	CTCGTCCGAGGGCAAAGGAATAGAGTAGAAGCCAGGAGCATAACCC	PCR primers for amplification of the downstream fragment of 06569
06569-DOWN-R	GTTTGCCAACTGGTCTGA	PCR primers for amplification of the downstream fragment of 06569
06569-ID-2335-F	TCCTCCAGCAATACCGAA	PCR primers for the identification of 06569 disruption
06569-ID-1746-R	GCAATACTCCGCCCCTTA	PCR primers for the identification of 06569 disruption
08672-UP-F	AGCCAGTGATAGGACGCA	PCR primers for amplification of the upstream fragment of 08672
08672-UP-R	CAAAATAGGCATTGATGTGTTGACCTCCCCTGTGGACCGAGTGAGA	PCR primers for amplification of the upstream fragment of 08672
08672-DOWN-F	CTCGTCCGAGGGCAAAGGAATAGAGTAGATTGTCATTGCCTTGTTTCG	PCR primers for amplification of the downstream fragment of 08672
08672-DOWN-R	GGGCGTTGGGTCTGTATC	PCR primers for amplification of the downstream fragment of 08672
08672-ID-2676-F	CCTGCCTCCCAACCTTTA	PCR primers for the identification of 08672 disruption
08672-ID-1635-R	CCAATGAGTGCGATAACAAA	PCR primers for the identification of 08672 disruption
09335-UP-F	TACGAGGTTTCTCACAGGA	PCR primers for amplification of the upstream fragment of 09335
09335-UP-R	CAAAATAGGCATTGATGTGTTGACCTCCGGGAGGGTTAGACAGGATA	PCR primers for amplification of the upstream fragment of 09335

09335-DOWN-F	CTCGTCCGAGGGCAAAGGAATAGAGTAGTGCCTGCTCCATCGTGAA	PCR primers for amplification of the downstream fragment of 09335
09335-DOWN-R	GTTGCTGTGACTGTCTACCA	PCR primers for amplification of the downstream fragment of 09335
09335-ID-3724-F	TTCCCAACAAAACACCAA	PCR primers for the identification of 09335 disruption
09335-ID-1733-R	CAAAAGGCAGCGTTAGAG	PCR primers for the identification of 09335 disruption
12768-UP-F	ATCCGTCACCACCACCCA	PCR primers for amplification of the upstream fragment of 12768
12768-UP-R	CAAAATAGGCATTGATGTGTTGACCTCCCGCCCAACAGAACAAACCAC	PCR primers for amplification of the upstream fragment of 12768
12768-DOWN-F	CTCGTCCGAGGGCAAAGGAATAGAGTAGGTTGGTGTTTCTTGGGTT	PCR primers for amplification of the downstream fragment of 12768
12768-DOWN-R	GTGTAACAGCAGCCTCATC	PCR primers for amplification of the downstream fragment of 12768
12768-ID-2723-F	ACAAGACCAGCAAAGCAT	PCR primers for the identification of 12768 disruption
12768-ID-1736-R	CCCTACAGAAAGTGGGAAAT	PCR primers for the identification of 12768 disruption
13650-UP-F	ACGACGGAGGCGTTACAG	PCR primers for amplification of the upstream fragment of 13650
13650-UP-R	CAAAATAGGCATTGATGTGTTGACCTCCCGGGGAACAAGGGAAGAG	PCR primers for amplification of the upstream fragment of 13650
13650-DOWN-F	CTCGTCCGAGGGCAAAGGAATAGAGTAGATCGGATAACACTGAACTAAG	PCR primers for amplification of the downstream fragment of 13650
13650-DOWN-R	TCTGTCCAACCTGATGAAA	PCR primers for amplification of the downstream fragment of 13650
13650-ID-2793-F	CCGAGAAACGGTTGATGA	PCR primers for the identification of 13650 disruption
13650-ID-1738-R	GCTTATGGCGACAGGAGTA	PCR primers for the identification of 13650 disruption
00226-UP-F	CTCATCGCCTTCATAAACA	PCR primers for amplification of the upstream fragment of 00226
00226-UP-R	CAAAATAGGCATTGATGTGTTGACCTCCTCCGAGTGGGGAATAGTG	PCR primers for amplification of the upstream fragment of 00226
00226-DOWN-F	CTCGTCCGAGGGCAAAGGAATAGAGTAGCTTCAGACCAAAACGAGC	PCR primers for amplification of the downstream fragment of 00226
00226-DOWN-R	GGGAGTCACAGTTACCAAA	PCR primers for amplification of the downstream fragment of 00226
00226-ID-2410-F	AAAGTAATCCACCTACAGAGC	PCR primers for the identification of 00226 disruption
00226-ID-1730-R	AATGGGTCCAGTCAACAG	PCR primers for the identification of 00226 disruption
09737-UP-F	CTATTCGTCTTTCGTGGTT	PCR primers for amplification of the upstream fragment of 09737
09737-UP-R	CAAAATAGGCATTGATGTGTTGACCTCCTCATTATTCCCGACACTTC	PCR primers for amplification of the upstream fragment of 09737

09737-DOWN-F	CTCGTCCGAGGGCAAAGGAATAGAGTAGCTCCCACATAAGCCAATC	PCR primers for amplification of the downstream fragment of 09737
09737-DOWN-R	CCGAGTAATGTAAGCCAAA	PCR primers for amplification of the downstream fragment of 09737
09737-ID-2258-F	TCTGCTATTGGTGGTGATG	PCR primers for the identification of 09737 disruption
09737-ID-1758-R	TCCCAGTTGCTACAGGTC	PCR primers for the identification of 09737 disruption
Actin-RT-F	ATCCACGTCACCACTTTCAA	PCR primers to amplify the *Actin* gene in quantitative real-time PCR assays
Actin-RT-R	TGCTTGGAGATCCACATTTG	PCR primers to amplify the *Actin* gene in quantitative real-time PCR assays
09737-RT-F	GCAAGTCGAGACTAGCGTTGC	PCR primers to amplify the *09737* gene in quantitative real-time PCR assays
09737-RT-R	CGACGAGTCTCAGGAGTGG	PCR primers to amplify the *09737* gene in quantitative real-time PCR assays
TRI1-F-RT	CATGGGCTTCGGTTACGGAG	PCR primers to amplify the *FgTRI1* gene in quantitative real-time PCR assays
TRI1-R-RT	GTCATCCTGTACCAAT	PCR primers to amplify the *FgTRI1* gene in quantitative real-time PCR assays
TRI4-F-RT	GATTGGAGGAGAACACTTGC	PCR primers to amplify the *FgTRI4* gene in quantitative real-time PCR assays
TRI4-R-RT	TGGAATTGCCTTGGGGTA	PCR primers to amplify the *FgTRI4* gene in quantitative real-time PCR assays
TRI5-F-RT	CTCGATACCGCCTCCATGAG	PCR primers to amplify the *FgTRI5* gene in quantitative real-time PCR assays
TRI5-R-RT	CTCCACTAGCTCAATTGAAC	PCR primers to amplify the *FgTRI5* gene in quantitative real-time PCR assays
TRI6-F-RT	AATGCCCTCAGTCAGCTCAA	PCR primers to amplify the *FgTRI6* gene in quantitative real-time PCR assays
TRI6-R-RT	ATCCGCCTATAGTGATCTCGC	PCR primers to amplify the *FgTRI6* gene in quantitative real-time PCR assays
TRI10-F-RT	GAGCGTTACTTGGCCTCTAG	PCR primers to amplify the *FgTRI10* gene in quantitative real-time PCR assays
TRI10-R-RT	TACCAAAGTAGCACTGGAAG	PCR primers to amplify the *FgTRI10* gene in quantitative real-time PCR assays
TRI101-F-RT	GAGTTCTTGGGCCAAGGTG	PCR primers to amplify the *FgTRI101* gene in quantitative real-time PCR assays
TRI101-R-RT	AACCAACGTACTGCGCATAC	PCR primers to amplify the *FgTRI101* gene in quantitative real-time PCR assays
